# Federally-Assisted Healthcare Coverage among Male State Prisoners with Chronic Health Problems

**DOI:** 10.1371/journal.pone.0160085

**Published:** 2016-08-01

**Authors:** David L. Rosen, Catherine A. Grodensky, Tara K. Holley

**Affiliations:** 1 Department of Medicine, Division of Infectious Diseases, University of North Carolina at Chapel Hill, Chapel Hill, North Carolina, United States of America; 2 Institute for Global Health and Infectious Diseases, University of North Carolina at Chapel Hill, Chapel Hill, North Carolina, United States of America; 3 Department of Behavioral Medicine, Duke University, Durham, North Carolina, United States of America; Johns Hopkins University Bloomberg School of Public Health, UNITED STATES

## Abstract

Prisoners have higher rates of chronic diseases such as substance dependence, mental health conditions and infectious disease, as compared to the general population. We projected the number of male state prisoners with a chronic health condition who at release would be eligible or ineligible for healthcare coverage under the Affordable Care Act (ACA). We used ACA income guidelines in conjunction with reported pre-arrest social security benefits and income from a nationally representative sample of prisoners to estimate the number eligible for healthcare coverage at release. There were 643,290 US male prisoners aged 18–64 with a chronic health condition. At release, 73% in Medicaid-expansion states would qualify for Medicaid or tax credits. In non-expansion states, 54% would qualify for tax credits, but 22% (n = 69,827) had incomes of ≤ 100% the federal poverty limit and thus would be ineligible for ACA-mediated healthcare coverage. These prisoners comprise 11% of all male prisoners with a chronic condition. The ACA was projected to provide coverage to most male state prisoners with a chronic health condition; however, roughly 70,000 fall in the “coverage gap” and may require non-routine care at emergency departments. Mechanisms are needed to secure coverage for this at risk group and address barriers to routine utilization of health services.

## Introduction

Prisoners have higher rates of chronic diseases such as substance dependence, mental health conditions and infectious disease, as compared to the general population.[1–3] The most recent national survey of prisoners (2011–2012) suggests that 50% of state and federal prisoners have a chronic medical condition.[1] Though limited, existing research suggests that prior to 2014, most released prisoners did not have health insurance in the 8–10 months after release. [4] Reasons for this lack of insurance have not been well documented, but are likely related to lack of employment (and employment-based insurance), limited perceived need among those who were young and healthy, other competing priorities such as securing food and housing, and difficulty enrolling in social safety net programs providing healthcare coverage. Lack of insurance was particularly prevalent among released men,[4] who comprised 89% of all released prisoners in 2013.[5]

Healthcare coverage rates among released prisoners may be improving with the roll-out of the Affordable Care Act (ACA).[6,7] The ACA provides several mechanisms to increase healthcare coverage. Two primary mechanisms include tax credits to help middle-income persons purchase private insurance through the state or federal “marketplaces” and the expansion of Medicaid eligibility criteria. Whereas Medicaid traditionally served impoverished adults who were aged, disabled, or had dependents, the ACA expanded criteria to provide coverage to low-income adults regardless of disability or dependents.

In 2012 the US Supreme Court ruled that states were not bound to expand Medicaid under the ACA.[8] In 2014, the first year of the ACA’s prescribed Medicaid expansion, 27 states expanded their eligibility criteria.[9] In these states, single adults (and families) with incomes equal to 138% of federal poverty level (FPL) or less were eligible to enroll in Medicaid. ACA subsidies for private insurance were available for those between 139% and 400% FPL. In non-expansion states, subsidies were available to those 101%-400% FPL, but Medicaid was not extended to those at 100% FPL ($11,290) or less, creating a “coverage gap.”[10]

Prior to the Court’s 2012 Medicaid ruling, Cuellar and Cheema projected that 57% of newly released US state prisoners could gain coverage under the ACA,[11] but these estimates assumed universal Medicaid expansion and did not account for enrollment in Medicare or “traditional” Medicaid (for the aged, disabled or those with dependents) for which automatic enrollment occurs in most states when qualifying for Social Security Administration (SSA) benefits, e.g. “Disability.”

We therefore undertook the current study, which accounts for the 2014 landscape of state Medicaid expansion policies and SSA-mediated benefits, to project post-release healthcare coverage rates—and the coverage gap—for male state prisoners with chronic health problems. We specifically focused on this population because of its large size, and because these inmates would likely require healthcare following release but have traditionally had low rates of healthcare coverage in the community.

## Materials and Methods

### Data source

Our analyses utilize data from the 2004 Survey of Inmates in State Correctional Facilities, which was conducted by the U.S. Census Bureau on behalf of the Bureau of Justice Statistics.[12] The survey was administered to a nationally representative sample of state prisoners using face-to-face interviews and included items assessing prisoners’ health conditions, pre-incarceration Social Security Administration (SSA) benefits, income, and other socio-demographic characteristics. The data and codebook are available online (www.icpsr.umich.edu). We provide the main survey items utilized in our analyses in the S1 Appendix.

### Study population

Our study population was composed of male state prisoners aged 18–64 years who reported a history of at least one of the 18 chronic health conditions assessed in the survey: hypertension, diabetes, heart disease, stroke, asthma, kidney disease, arthritis, hepatitis, cirrhosis, cancer, human immunodeficiency virus (HIV), paralysis, depression, anxiety, post-traumatic stress disorder (PTSD), bipolar disorder, a psychotic disorder, or a personality disorder.

### Primary Analysis

We described the socio-demographic characteristics and prevalence of reported chronic health conditions among the study population. We then used the joint distributions of reported SSA benefits, income, and health conditions to project the number of male prisoners with a chronic condition who would be eligible for healthcare coverage at release. We estimated the number with a history of SSA benefits in the month prior to incarceration and assumed that SSA-mediated insurance could be resumed post-release. Among those without SSA benefits in the month prior to incarceration, we determined eligibility for Medicaid and tax credits by comparing prisoners’ pre-incarceration annual income (i.e. projected from income the month prior to incarceration, adjusted for inflation using the US Bureau of Labor Statistics Employment Cost Index[13]) to the 2014 Medicaid/tax credit FPL criteria described above. Sixteen percent were missing income data. To impute missing income values, we jointly stratified prisoners by age (group), race, and education level, and assigned those with missing incomes the median income value of prisoners within their same stratum who had non-missing data. For all and each chronic condition(s), we estimated the number of prisoners eligible for healthcare coverage in Medicaid expansion and non-expansion states. We applied the sampling weights included in the dataset so that results would reflect the total population of adult, non-elderly US male prisoners with a history of chronic medical condition. All analyses, including the sensitivity analyses described below, were conducted using SAS version 9.4 (Cary, North Carolina). Weighted population means and frequencies and corresponding 95% confidence intervals (CIs) were estimated using proc surveymeans and proc surveyfreq.

### Sensitivity analyses

Reported income in the month prior to arrest was recorded using categorical responses (e.g. No income, $1–199, $200–399… . $5000–7499, $7500 or more). In our main analysis, we assume respondents’ pre-incarceration monthly income was the mid-point value of the selected range (e.g. inmates reporting an income of $1–199 were assigned an income of $100). Projected income was estimated as the monthly income, multiplied by 12, and adjusted for inflation to 2014 dollars. We conducted two sets of sensitivity analyses to determine the number of prisoners in the coverage gap by assuming that respondents’ true income was at the lowest and highest value of each selected category. Several studies suggest that prisoners may encounter a loss of earning or “wage penalty” following an incarceration. In line with the earlier analysis by Cuellar and Cheema, we examined the effect of a 15% “wage penalty,” on our results, which we applied to our “low,”“mid-point” and “high” analyses resulting in six estimates of the number of male state prisoners who would be within the coverage gap at release. This study was deemed non-human subjects research by the Institutional Review Board of the University of North Carolina at Chapel Hill.

## Results

There were 643,290 male prisoners aged 18–64 with a chronic health condition (Table 1). Briefly, 60% were non-White, 11% were aged over 50 years, and 34% had neither completed high school nor earned a high school equivalency certificate (GED). The median annual pre-incarceration income, adjusted to 2014 dollars, was $18,531. Seven percent received SSA benefits in the month prior to incarceration, and prisoners with any chronic condition were nearly evenly distributed across expansion (51%) and non-expansion (49%) states (Table 1).

**Table 1:** Characteristics of male US state prisoners with a chronic health condition (N = 643,290)*.

		Lower	Upper
	%	95% CI	95% CI
Age (years)			
18–50	88.7	86.2	91.3
51–64	11.3	10.1	12.5
Race			
White	40.0	37.5	42.5
Black	37.8	35.7	39.8
Other	22.3	20.8	23.7
Education			
No HS or GED	33.9	32.1	35.7
HS or GED	52.5	50.4	54.6
post HS or GED	13.3	12.3	14.2
Median annual Income ($)**	18,531	17,897	19,165
Homeless at arrest			
Yes	2.2	1.8	2.7
Received SSA benefits in the month prior to arrest			
Yes	7.0	6.2	7.8
Ever received SSA benefits			
Yes	12.3	11.3	13.4
Medicaid expansion state			
Yes	50.9	46.6	55.2
Chronic condition			
Hypertension	35.2	33.4	36.9
Diabetes	7.5	6.7	8.2
Heart disease	15.2	14.1	16.3
Stroke	7.4	6.6	8.3
Asthma	24.1	22.8	25.5
Kidney disease	9.8	8.9	10.7
Arthritis	25.5	23.8	27.1
Hepatitis	15.8	14.5	17.2
Cirrhosis	3.0	2.5	3.4
Cancer	2.8	2.3	3.2
HIV	2.0	1.5	2.5
Paralysis	10.5	9.5	11.4
Depression	30.8	28.6	33.0
Anxiety Disorder	11.0	9.8	12.2
PTSD	8.8	7.9	9.8
Bipolar	15.0	13.7	16.3
Psychotic disorder	7.7	6.7	8.7
Personality Disorder	10.0	8.9	11.0

* weighted estimates, based on 2004 survey data

** Respondents reported income by selecting one of several income categories (e.g. No income, $1–100, $200–400, etc.) for the month prior to their incarceration.

For the main analysis, we assigned inmates to the average of the lowest and highest value within selected category. The average values were multiplied by 12 to estimate annual income and adjusted for inflation to 2014 dollars. For the 16% missing income data, we imputed values as the median income of prisoners of a similar age, race, and education Abbreviations: Confidence interval (CI); High School (HS) or General Educational Development/high school degree equivalency (GED); Post-traumatic stress disorder (PTSD);Social Security Administration (SSA)

**Fig 1:**
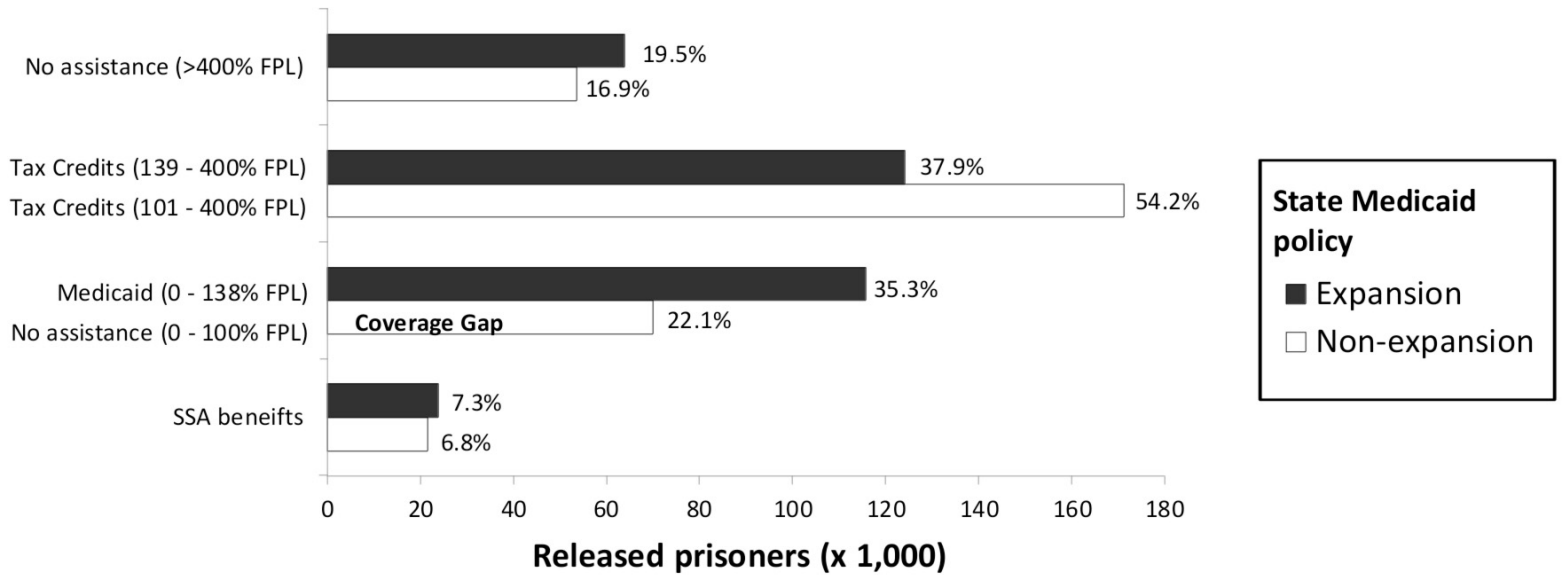
Projected healthcare coverage assistance for released male US state prisoners with chronic health conditions in Medicaid expansion and non-expansion states. Estimates to right of bars reflect percentage of prisoners by state Medicaid policy; FPL: 2014 Federal poverty level ($11,670); SSA: Social Security Administration.

In Medicaid expansion and non-expansion states, 20% and 17% of prisoners, respectively, were projected to earn ≥400% FPL and would thus not be eligible for Medicaid or tax credits, and about 7% of prisoners in expansion and non-expansion states had a history of SSA benefits, implying future Medicaid or Medicare coverage. In expansion states, 73% of prisoners qualified for Medicaid or tax credits. In non-expansion states, 54% qualified for tax credits, but 22% (n = 69827) were projected to have incomes ≤ 100% FPL and would not qualify for any support (Fig 1), placing them in the coverage gap.

Among the prisoners in the coverage gap, the ten most frequently reported conditions in order of descending frequency were hypertension (n = 27245), depression, arthritis, asthma, hepatitis, heart disease, bipolar, kidney disease, personality disorder, and anxiety disorder (n = 7283) (Table 2).

**Table 2:** Projected sources of healthcare coverage assistance for released US male state prisoners with chronic health conditions in Medicaid expansion and non-expansion states, by condition.

**Expansion**			Medicaid		Tax credits		No assistance		
	SSA benefits		(0–138% FPL)		(139–400% FPL)		>400% FPL		Total
	n	%	n	%	n	%	n	%	n
Hypertension	9106	8.8	37716	36.4	37715	36.4	19153	18.5	103690
Diabetes	2379	9.6	9814	39.6	8050	32.5	4529	18.3	24772
Heart disease	5509	11.3	16833	34.4	18366	37.5	8242	16.8	48950
Stroke	3564	14.1	9036	35.7	8364	33.1	4321	17.1	25285
Asthma	6241	7.2	29544	34.3	34589	40.1	15857	18.4	86231
Kidney	3492	12.5	8055	28.8	10576	37.8	5856	20.9	27979
Arthritis	8368	10.6	27191	34.6	29042	36.9	14032	17.8	78633
Hepatitis	5315	9.7	19621	36.0	20898	38.3	8708	16.0	54542
Cirrhosis	788	8.5	2538	27.5	4224	45.7	1693	18.3	9243
Cancer	1510	15.8	2775	29.0	3067	32.1	2214	23.1	9566
HIV	1506	27.5	2218	40.5	1157	21.1	592	10.8	5473
Paralysis	4658	13.0	11577	32.3	12713	35.4	6921	19.3	35869
Depression	11362	11.4	34705	35.0	35437	35.7	17761	17.9	99265
Anxiety	4662	13.1	12999	36.6	12147	34.2	5744	16.2	35552
PTSD	4014	13.8	9628	33.2	9772	33.7	5576	19.2	28990
Bipolar	6694	13.7	15405	31.5	16702	34.2	10051	20.6	48852
Psychotic	5838	23.5	9394	37.8	6366	25.6	3277	13.2	24875
Personality	4177	12.7	11246	34.1	10077	30.5	7501	22.7	33001
Any condition	23773	7.3	115679	35.3	124050	37.9	63868	19.5	327370
**Non-expansion**			No support		Tax credits		No assistance		
	SSA benefits		(0–100% FPL)		(101–400% FPL)		>400% FPL		Total
	n	%	n	%	n	%	n	%	n
Hypertension	9367	7.6	27245	22.2	66529	54.2	19508	15.9	122649
Diabetes	2274	9.7	4953	21.2	12396	53.1	3729	16.0	23352
Heart disease	5842	12.0	11270	23.2	24881	51.2	6625	13.6	48618
Stroke	3561	15.9	5101	22.7	9865	44.0	3914	17.4	22441
Asthma	4510	6.5	16793	24.4	36839	53.5	10745	15.6	68887
Kidney	3061	8.7	8973	25.6	17651	50.4	5364	15.3	35049
Arthritis	8240	9.7	19009	22.3	42276	49.7	15580	18.3	85105
Hepatitis	2870	6.1	11361	24.1	27230	57.7	5703	12.1	47164
Cirrhosis	1168	11.8	2043	20.6	5169	52.2	1523	15.4	9903
Cancer	1070	13.2	1625	20.0	3382	41.7	2040	25.1	8117
HIV	668	9.0	1233	16.6	4941	66.6	579	7.8	7422
Paralysis	3954	12.6	6986	22.3	13727	43.8	6682	21.3	31349
Depression	8091	8.2	21202	21.5	53047	53.7	16475	16.7	98815
Anxiety	3661	10.4	7283	20.7	18009	51.1	6281	17.8	35234
PTSD	3023	10.9	5743	20.7	14293	51.5	4717	17.0	27776
Bipolar	4374	9.2	8996	18.9	25158	52.9	8994	18.9	47522
Psychotic	3816	15.3	6409	25.8	11028	44.3	3631	14.6	24884
Personality	2656	8.5	7585	24.4	15862	50.9	5046	16.2	31149
Any condition	21427	6.8	69827	22.1	171159	54.2	53507	16.9	315920

% represent row percentages

Abbreviations: 2014 Federal poverty level (FPL); Social Security Administration (SSA), Post-traumatic Stress Disorder (PTSD)

**Fig 2:**
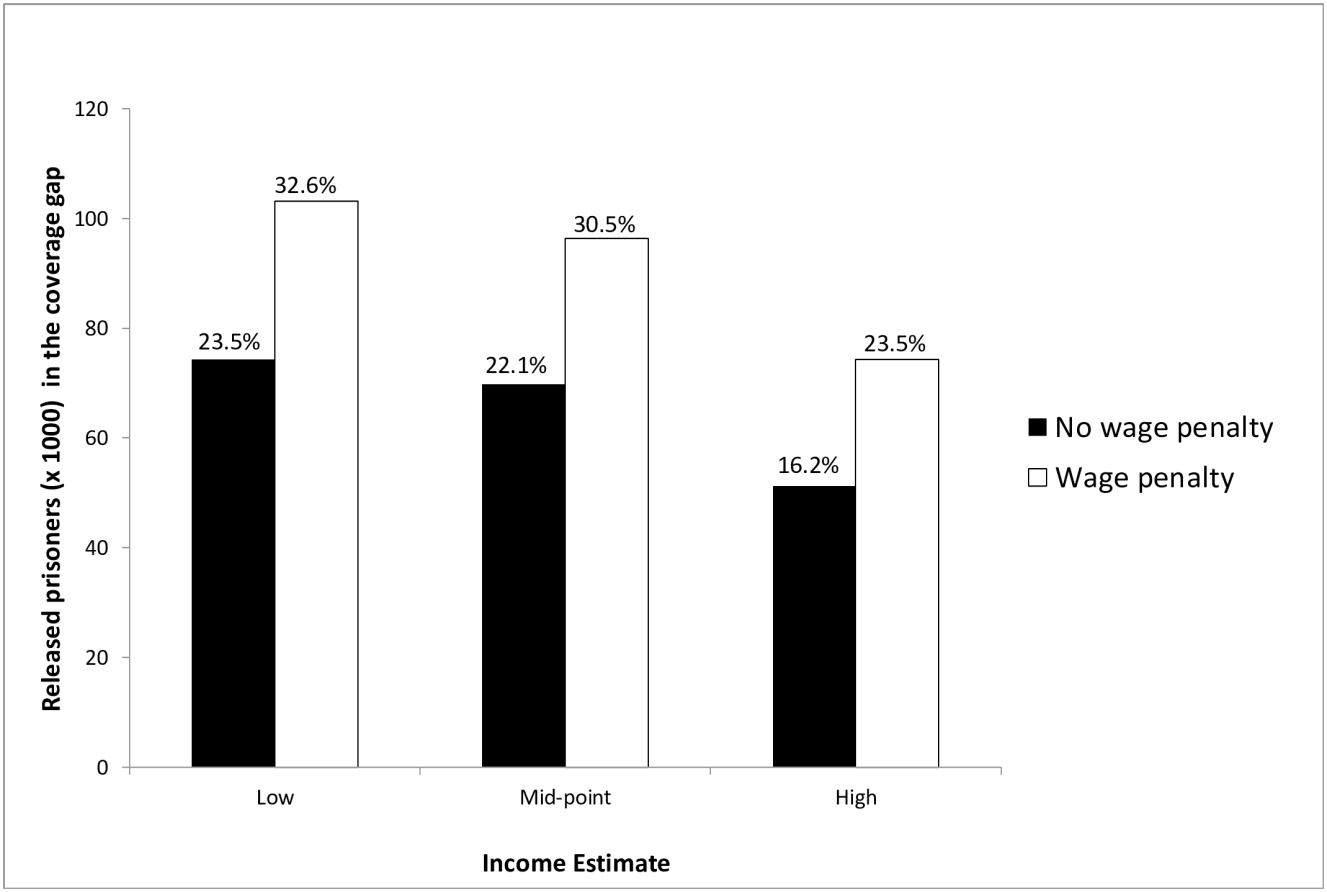
Sensitivity analysis projecting the number and percentage of male US state prisoners with a history of chronic medical conditions incarcerated in a Medicaid non-expansion state who will be in the "coverage gap"* at release. *In non-expansion states, subsidies are available to those with incomes 101%-400% FPL, but Medicaid was not extended to those at or below 100% FPL, creating a “coverage gap” among impoverished persons. **Respondents reported income by selecting one of several income categories (e.g. No income, $1–100, $200–400, etc.) for the month prior to their incarceration. We estimated monthly income based on the lowest and highest value and their average (i.e. "Mid-point") in the selected category. All monthly estimates were multiplied by 12 to estimate annual income and adjusted for inflation to 2014 dollars. Estimates were compared to 2014 ACA income guidelines to determine the percentage in the "coverage gap." ***Some evidence suggests that released prisoners earn less than their pre-incarceration wages. As with an earlier study[11], we applied a 15% wage penalty to the Low, Middle, and High pre-incarceration income estimates to account for this wage reduction in determining the percentage of prisoners in the "coverage gap."

In the primary analysis, prisoners in the coverage gap represented 11% of all prisoners in our target population (and 22% of those in non-expansion states). Across sensitivity analyses, which addressed uncertainty in prisoners’ post-release income, the number of prisoners in the coverage gap ranged from 51,254 (High income estimate, no wage penalty) to 103,128 (Low income estimate, 15% wage penalty), suggesting that 16–32% of prisoners in non-expansion states (Fig 2), and 8–16% of prisoners in our total population would be in the coverage gap.

## Discussion

Although the ACA could have a substantial impact on access to healthcare for released prisoners, previously published estimates of the number of prisoners eligible for healthcare coverage at release do not account for states that opted not to expand Medicaid. In this analysis, we accounted for the 2014 landscape of Medicaid expansion policies and estimated healthcare coverage eligibility under the ACA among male state prisoners with chronic health conditions. We also refined the estimates by accounting for inmates eligible for coverage under SSA benefits.

Our primary analysis suggests that about sixty-nine thousand male prisoners with selected chronic conditions would be ineligible for healthcare coverage under the ACA because of states that chose not to expand their Medicaid coverage to impoverished adults without disability or dependents. This population in the coverage gap represents 11% of all male prisoners aged 18–64 with selected chronic conditions, and based on our sensitivity analysis, the gap would not be expected to be lower than 8% or higher than 16%. In the national context, these results suggest that through Medicaid expansion or tax credits, the ACA can provide insurance to the majority of impoverished male prisoners with chronic health conditions in need of coverage.

Nevertheless, interventions are needed for the roughly 50,000 to 100,000 men in the coverage gap, most of whom are released from prison and returning to their home communities after serving one or two years. Clearly universal Medicaid expansion could further improve coverage rates, but Medicaid expansion remains politically contentious. In the absence of universal Medicaid expansion, an important point of access to healthcare coverage for those with debilitating conditions is through SSA benefits (such as “disability”), which can result in Medicare or Medicaid enrollment. We found that among those with a chronic condition (in both expansion and non-expansion states), only 7% had a history of SSA benefits in the month prior to arrest. These percentages likely underestimate the true proportion of prisoners who could qualify for SSA benefits if prisoners were evaluated thoroughly. The extent to which prison systems currently facilitate enrollment in SSA benefits (i.e., assist with applications) in preparation for prisoners’ release is unclear, but data from our 2011–2012 survey suggest that SSA enrollment assistance was uncommon.[14]

Released prisoners in the coverage gap may be at increased risk for relying on emergency departments (ED) for routine care. In a retrospective cohort study in Rhode Island of over 6,000 prisoners released over a two year period, at least 24% had one or more documented emergency department visits within the first year post-release.[15] ED use may be more common among those with serious chronic conditions. In a Connecticut study of prisoners with HIV, 56% (85/151) had an ED visit in the first year post-release.[16] While neither of these studies reported cost, existing evidence suggests that for ambulatory care sensitive conditions, charges and payments for ED care are about two times those for outpatient care (for similar conditions and treatment),[17] suggesting that released prisoners in the coverage gap may have little choice when seeking care but to utilize high-cost health services.

Even for released prisoners who establish healthcare coverage, challenges remain. Beyond issues common to prisoner re-entry (e.g. establishing employment, securing housing, and addressing substance use), those with Medicaid may face particular barriers to healthcare access. Results from a 2013 survey conducted by the Department of Health and Human Services Office of Inspector General (OIG) in 32 states with Managed Care Organization (MCO)-facilitated Medicaid, found that greater than half of providers listed by the MCO as participating in Medicaid could not be found at their listed location or were not accepting new patients; among providers who were accepting new patients, greater than 25% had wait times of one month or longer. The OIG concluded there were “significant vulnerabilities in provider availability, which is a key indicator for access to care.”[18]

In addition to issues of access, released prisoners who obtain healthcare coverage, regardless of the mechanism, may face discrimination in accessing care. For example, in a small but important study, Frank et al. found that 42% (73/172) of male parolees felt discriminated against in a health care setting because of their criminal record.[19] Such discrimination likely decreases service use, but more research in this area is needed.

To address these problems of access and discrimination, the Transitions Clinic Network has been developed specifically for former prisoners. This network is based on the Transition Clinic model, which includes healthcare delivered by providers experienced with criminal justice-involved populations and a case manager with a personal history of incarceration who coordinates resources and provides guidance in chronic disease management. In a 2007 randomized trial of 200 recently released prisoners, half received care at a Transitions Clinic (TC) and half received an “expedited primary care appointment at another safety net clinic.” Investigators found that the rate of primary care use was similar across groups but there was less utilization of ED visits among the TC group.[20] Funded under a Centers for Medicare and Medicaid Services Innovation award, the TC network is providing care for Medicaid-eligible released prisoners and is now being evaluated for its ability to lower costs, improve access to healthcare, and improve health. Results will be forthcoming.

The current analysis has several limitations and our estimates should be interpreted in the proper context—they provide a plausible set of estimates for healthcare coverage eligibility among state prisoners with chronic conditions residing in Medicaid expansion and non-expansion states. However, as with the Cuellar and Cheema study, these estimates are based on data from a 2004 survey. Although existing data suggests that the size of the prison population and level of morbidity has changed little over time,[1,21,22] joint data on prisoners’ morbidity, income/wealth, and health care eligibility were not available for 2014. Similarly, although the proportion of inmates eligible for Medicare (i.e. aged ≥ 65 years) grew between 2004 and 2014, the increase was sufficiently modest (~ 1% point) to not incorporate into our analysis. Additionally, our estimates are based on prisoners’ disclosure of health status and income, both of which may be biased by self-report. Nevertheless, our sensitivity analyses of reported income provided plausible bounds for the effect of reported income on the number of prisoners in the coverage gap. Another possible limitation is the probable under- and over-reporting of conditions. Although thorough, the 2004 survey questionnaire did not include all possible health outcomes, and we chose to focus our analysis on inmates with a history of health conditions generally considered to be chronic, as these inmates are likely to require healthcare following release. Further, we focused our analysis on men because men comprise the vast majority of state prisoners and men and women have different patterns of health conditions, income, and healthcare coverage. With these differences in mind, a similar set of analyses among women prisoners are planned for the future.

We note that items informing Medicaid and subsidy eligibility such as family situation and sentence length were addressed in the 2004 survey, but we found the survey items of insufficient specificity to inform our analysis. Accordingly, our estimates are based on eligibility criteria for individuals and not for parents/families, and our analysis assumes that all prisoners will eventually be released. In fact, greater than 95% are released, most within 2–3 years.[22] Also, a few non-expansion states have extended Medicaid coverage to populations outside of the ACA framework.[23] Typically these expansions cover relatively small populations with restricted benefits as compared to full Medicaid expansion, and none are targeted at prisoners specifically; accordingly, this information was not integrated into our analysis. Similarly, our analysis does not account for other sources of healthcare coverage such as hospital charity care.

The next Bureau of Justice Statistics nationally representative survey of prisoners to include information on health conditions, income, entitlements and healthcare coverage is scheduled for administration in 2015–16; based on past BJS surveys, we anticipate that these data will not be released until 2019 or later. Until that time, our data may represent the best national estimates for released prisoners’ healthcare coverage eligibility.

## Conclusion

In conclusion, we estimate that about approximately 70,000 male prisoners with a history of chronic health problems fall within the healthcare coverage gap. Although existing research suggests that those without coverage will have inadequate healthcare use and poor health, more research is needed to delineate these effects. At the same time, future work should leverage mechanisms to improve healthcare access and health for this marginalized population. Although healthcare reform remains a contentious issue, there is growing support across the political spectrum that more resources—including access to healthcare—are needed if prisoners are to successfully re-integrate themselves in the communities to which they return. Debates over healthcare reform should not impede this important work.

## Supporting Information

S1 Appendix Survey items utilized in the analysis of healthcare coverage elgibility among US male state prisoners with chronic health conditions. (PDF)

## References

[1] Maruschak LM, Berzofsky M, Unangst J (2015) Medical Problems of State and Federal Prisoners and Jail Inmates, 2011–12. Washington, DC: US Department of Justice, Bureau of Justice Statistics. NCJ 248491 NCJ 248491.

[2] Wilper AP, Woolhandler S, Boyd JW, Lasser KE, McCormick D, et al (2009) The health and health care of US prisoners: results of a nationwide survey. Am J Public Health 99: 666–672. 10.2105/AJPH.2008.144279 19150898 PMC2661478

[3] Binswanger IA, Krueger PM, Steiner JF (2009) Prevalence of chronic medical conditions among jail and prison inmates in the USA compared with the general population. J Epidemiol Community Health 63: 912–919. 10.1136/jech.2009.090662 19648129

[4] Mallik-Kane K, Visher CA (2008) Health and Prisoner Reentry: How Physical, Mental, and Substance Abuse Conditions Shape the Process of Reintegration. Washington, DC: Urban Institue 1–68 p.

[5] Carson EA, Mulako-Wangota J (2015) Count of total releases. Generated using the Corrections Statistical Analysis Tool (CSAT)—Prisoners at www.bjs.gov. Bureau of Justice Statistics.

[6] Blair P, Greifinger R, Stone TH, Somers S (2011) Increasing access to health insurance coverage for pre-trial detainees and individuals transitioning from correctional facilities under the patient protection and afffordable care act. Avauilable: http://www.cochs.org/files/ABA/aba_final.pdf. Accessed 2013 May 10.

[7] Rich JD, Chandler R, Williams BA, Dumont D, Wang EA, et al (2014) How Health Care Reform Can Transform The Health Of Criminal Justice-Involved Individuals. Health Aff (Millwood) 33: 462–467. 24590946 10.1377/hlthaff.2013.1133 PMC4034754

[8] National Federation of Independent Business v. Sebelius, 567 U.S. ___ (2012) 132 S.Ct 2566. 23488086

[9] Kaiser Family Foundation (2014) State activity around expanding medicaid under the affordable care act.

[10] Garfield R, Damico A, Stephens J, Rouhani S (2014) The Coverage Gap: Uninsured Poor Adults in States that Do Not Expand Medicaid—An Update In: The Henry J. Kaiser Family Foundation, editor. The Kaiser Commission on Medicaid and the Uninsured. pp. 1–8.

[11] Cuellar AE, Cheema J (2012) As roughly 700,000 prisoners are released annually, about half will gain health coverage and care under federal laws. Health Aff (Millwood) 31: 931–938. 22566431 10.1377/hlthaff.2011.0501

[12] US Department of Justice, Bureau of Justice Statistics (2007) The 2004 Survey of Inmates in State Correctional Facilities Codebook (ICPSR 4572). ICPSR04572-v1. Ann Arbor, MI: Inter-university Consortium for Political and Social Research [distributor], 2007-02-28. 10.3886/ICPSR04572.v1

[13] US Bureau of Labor Statistics (2015) Contiunous Occupaational and Industry Series, September 1975- September 2015 (December 2005 = 100). Employment cost Index Historical Listing—Volume V. Available: http://www.bls.gov/web/eci/ecicois.pdf. pp. 1–68.

[14] Rosen DL, Dumont DM, Cislo AM, Brockmann BW, Traver A, et al (2014) Medicaid policies and practices in US state prison systems. Am J Public Health 104: 418–420. 10.2105/AJPH.2013.301563 24432881 PMC3953759

[15] Frank JW, Andrews CM, Green TC, Samuels AM, Trinh TT, et al (2013) Emergency department utilization among recently released prisoners: a retrospective cohort study. BMC Emerg Med 13: 16 10.1186/1471-227X-13-16 24188513 PMC3818565

[16] Meyer JP, Qiu J, Chen NE, Larkin GL, Altice FL (2012) Emergency department use by released prisoners with HIV: an observational longitudinal study. PLoS One 7: e42416 10.1371/journal.pone.0042416 22879972 PMC3411742

[17] Galarraga JE, Mutter R, Pines JM (2015) Costs Associated with Ambulatory Care Sensitive Conditions Across Hospital-based Settings. Acad Emerg Med. 10.1111/acem.12579 25639774

[18] Office of Inspector General (2014) Access to care: provider availability in medicaid managed care. Washington, D.C.: Department of Health and Human Services Available: http://oig.hhs.gov/oei/reports/oei-02-13-00670.pdf. Accessed 2015 Feb 3.

[19] Frank JW, Wang EA, Nunez-Smith M, Lee H, Comfort M (2014) Discrimination based on criminal record and healthcare utilization among men recently released from prison: a descriptive study. Health Justice 2: 6 25642407 10.1186/2194-7899-2-6 PMC4308970

[20] Wang EA, Hong CS, Shavit S, Sanders R, Kessell E, et al (2012) Engaging individuals recently released from prison into primary care: a randomized trial. Am J Public Health 102: e22–29. 10.2105/AJPH.2012.300894 PMC3482056 22813476

[21] Harrison PM, Beck AJ (2005) Prisoners in 2004. Washington, DC: US Department of Justice, Bureau of Justice Statistics. NCJ 210677 NCJ 210677.

[22] Carson EA (2014) Prisoners in 2013. Washington, DC: US Department of Justice, Bureau of Justice Statistics. NCJ 247282 NCJ 247282.

[23] Rudowitz R, Artiga S, Arguello R (2013) Appendix B: Waivers for Childless Adults: Income Eligibility Limits as a Percent of the FPL, Waiver Timing and Medicaid Expansion Status. A Look at Section 1115 Medicaid Demonstration Waivers Under the ACA: A Focus on Childless Adults Kaiser Family Foundation. Availabe at http://kff.org/report-section/section-1115-medicaid-demonstration-waivers-appendix. Accessed 2015 Apr 30.

