# Supplementary material for: Federally-Assisted Healthcare Coverage among Male State Prisoners with Chronic Health Problems
Source: PLoS One. 2016 Aug 1;11(8):e0160085. doi: 10.1371/journal.pone.0160085 (PMC4968827; doi:10.1371/journal.pone.0160085)
Supplement: S1 Appendix — (PDF) [file pone.0160085.s001.pdf]

S1 Appendix. Survey items utilized in the analysis of healthcare coverage elgibility among US male state prisoners with chronic health conditions

Domain	Variable	Item
Socio-demographics	V0004	SEX<br>Location: 14-14 (width: 1; decimal: 0)<br>Interval:discrete<br>Question: S1Q1<br>Label Value<br>Male 1<br>Female 2<br>Blank 9 (M)
Socio-demographics	V0014	AGE (ALLOCATED)<br>Location: 35-37 (width: 3; decimal: 0)<br>Interval: continuous<br>Range of Valid Data Values: 000 thru 996<br>Question: AGE
Socio-demographics	V2982	RACEHISP: Race and Hispanic Origin of Inmate<br>Location: 8840-8840 (width: 1; decimal: 0)<br>Interval: discrete<br>Question: RACEHISP: Race and Hispanic Origin of Inmate Question:<br>Value Label<br>1 White non-Hispanic<br>2 Black non-Hispanic<br>3 Hispanic<br>4 American Indian, Alaska Native non-Hispanic<br>5 Asian, Pacific Islander, Native Hawaiian non-Hispanic<br>6 Multiple Races reported, non-Hispanic<br>8 Other, Uncategorized - Missing
Socio-demographics	V1740	S7Q1A: BEFORE ADMISSION, HIGHEST GRADE OF SCHOOL ATTENDED<br>Location: 4479-4480 (width: 2; decimal: 0)<br>Interval: discrete<br>Question: S7Q1A: This group of questions concerns your education, employment, and income.S7Q1A: This group of questions concerns your education, employment, and income. Before your admission on [MOST RECENT ADMISSION DATE), what was the highest grade of school that you ever attended?<br>[Response options not shown]
Socio-demographics	V1777	S7Q6A_1: BEFORE ARREST LIVING IN A -<br>Location: 4546-4547 (width: 2; decimal: 0)<br>Interval: discrete<br>Question: S7Q6A_1: Before your arrest [CONTROLLING ARREST DATE], were you living in a -<br>Value Label<br>01 House?<br>02 Apartment?<br>03 Trailer or mobile home?<br>04 Rooming-house, hotel or motel?<br>05 On the street or in a homeless shelter?<br>In a group living situation or institution, such as a hospital, halfway house, recovery room, dormitory, etc.<br>06<br>07 In another type of housing?<br>97 Don't know<br>98 Refused<br>99 (M) Blank

Pre-incarceration income	V1857	S7Q11C: MONTH BEFORE ARREST MONTHLY INCOME<br>Location: 4679-4680 (width: 2; decimal: 0)<br>Interval: discrete<br>Question: S7Q11C: Which category on this card represents your personal monthly income from ALL sources for the month before your arrest, that is, from [MONTH] 1st to [MONTH] [28-31], [YEAR].<br>Question:<br>Value Label<br>00 No income<br>01 $1 - 199<br>02 200 - 399<br>03 400 - 599<br>04 600 - 799<br>05 800 - 999<br>06 1,000 - 1,199<br>07 1,200 - 1,499<br>08 1,500 - 1,999<br>09 2,000 - 2,499<br>10 2,500 - 4,999<br>11 5,000 - 7,499<br>12 7,500 or more<br>97 Don't know<br>98 Refused<br>99 (M) Blank
Pre-incarceration benefits	V1847	S7Q11A1_2: MONTH BEFORE ARREST RECEIVED INCOME FROM SSI/SSDI<br>Location: 4669-4669 (width: 1; decimal: 0)<br>Interval: discrete<br>Question: S7Q11A1_2: During the month before your arrest, from [MONTH] 1st to [MONTH] [28-31], [YEAR], did you personally receive any money/income from - Social security or supplemental security income (SSI), or Social Security Disability Insurance (SSDI)?<br>Question:<br>Value Label<br>1 Yes<br>2 No<br>7 Don't know<br>8 Refused<br>9 (M) Blank
Pre-incarceration benefits	V1858	S7Q11D: RECEIVED INCOME FROM SOCIAL SECURITY<br>Location: 4681-4681 (width: 1; decimal: 0)<br>Interval: discrete<br>Question: S7Q11D: Have you ever received income from Social Security as a result of a retirement, survivors, or disability benefit?<br>Question:<br>Value Label<br>1 Yes<br>2 No<br>7 Don't know<br>8 Refused<br>9 (M) Blank
State	V1057	S5Q15A_ST: AT ARREST - RESIDENCE (STATE)<br>Location: 2999-3000 (width: 2; decimal: 0)<br>Interval: discrete<br>Question: S5Q15A_ST: At the time of your arrest [CONTROLLING ARREST DATE], in what city or place did you live? (State)<br>[Options not shown]
Health Conditions	V2289	S9Q4C: RESULT OF LAST (HIV) TEST<br>Location: 5243-5243 (width: 1; decimal: 0)<br>Interval: discrete<br>Question: S9Q4C: What was the result of the last test you had?<br>Value Label<br>1 Positive (infected with the AIDS virus)<br>2 Negative (not infected)<br>3 Result not available yet<br>7 Don't know<br>8 Refused<br>9 (M) Blank

Health Conditions	V2319	S9Q6A: EVER HAD CANCER<br>Location: 5289-5289 (width: 1; decimal: 0)<br>Interval: discrete<br>Question: S9Q6A: Have you EVER had any type of cancer?<br>Value Label<br>1 Yes<br>2 No<br>7 Don't know<br>8 Refused<br>9 (M) Blank
Health Conditions	V2335	S9Q6B: EVER EXPERIENCED PARALYSIS<br>Location:5317-5317 (width: 1; decimal: 0)<br>Interval: discrete<br>S9Q6B: Have you ever been paralyzed or unable to move your legs, arms, or other areas of your body? Do not include times when you may have been held down or tied up.<br>Value Label<br>1 Yes<br>2 No<br>7 Don't know<br>8 Refused<br>9 (M) Blank
Health Conditions	V2336	S9Q6C: EVER HAD HYPERTENSION<br>Location:5320-5320 (width: 1; decimal: 0)<br>Interval: discrete<br>S9Q6C: Have you ever had high blood pressure or hypertension?<br>Value Label<br>1 Yes<br>2 No<br>7 Don't know<br>8 Refused<br>9 (M) Blank
Health Conditions	V2341	S9Q6E: EVER HAD DIABETES<br>Location: 5326-5326 (width: 1; decimal: 0)<br>Interval: discrete<br>Question: S9Q6E: Have you ever had diabetes or high blood sugar? Question:<br>Label Value<br>Yes 1<br>No 2<br>Don't know 7<br>Refused 8<br>Blank 9 (M)
Health Conditions	V2347	S9Q6F: EVER HAD HEART PROBLEMS<br>5329-5329 (width: 1; decimal: 0) Location:<br>discrete Interval:<br>Question S9Q6F: Have you ever had problems with your heart? Question:<br>Label Value<br>Yes 1<br>No 2<br>Don't know 7<br>Refused 8<br>Blank 9 (M)
Health Conditions	V2360	S9Q6G: EVER HAD KIDNEY PROBLEMS<br>5350-5350 (width: 1; decimal: 0) Location:<br>discrete Interval:<br>Question S9Q6G: Have you ever had problems with your kidneys? Question:<br>Label Value<br>Yes 1<br>No 2<br>Don't know 7<br>Refused 8<br>Blank 9 (M)

Health Conditions V2363 S9Q6H: EVER HAD ARTHRITIS OR RHEUMATISM
5353-5353 (width: 1; decimal: 0) Location:
discrete Interval:
Quesiton S9Q6H: Have you ever had arthritis or rheumatism? Question:
Label Value
Yes 1
No 2
Don't know 7
Refused 8
Blank 9 (M)

Health Conditions V2366 S9Q6I: EVER HAD ASTHMA
(width: 1; decimal: 0) Location: 5356-5356
discrete Interval:
Question S9Q6I: Have you ever had asthma? Question:
Label Value
Yes 1
No 2
Don't know 7
Refused 8
Blank 9 (M)

Health Conditions V2369 S9Q6J: EVER BEEN TOLD HAVE CIRRHOSIS
Location: 5359-5359 (width: 1; decimal: 0)
Interval: discrete
Question S9Q6J: Have you ever been told that you have Cirrhosis of the liver? Question:
Label Value
Yes 1
No 2
Don't know 7
Refused 8
Blank 9 (M)

Health Conditions V2372 S9Q6K: EVER BEEN TOLD HAVE HEPATITIS
Location: 5362-5362 (width: 1; decimal: 0)
Interval: discrete
Question: S9Q6K: Have you ever been told that you have Hepatitis?
Value Label
1 Yes
2 No
7 Don't know
8 Refused
9 (M) Blank

Health Conditions V2401 S9Q9A_1: EVER DIAGNOSED - A DEPRESSIVE DISORDER
Location: 5391-5391 (width: 1; decimal: 0)
Interval: discrete
Question: S9Q9A_1: Have you ever been told by a mental health professional, such as a psychiatrist or psychologist, that you had - A depressive disorder
Label Value
Yes 1
No 2
Don't know 7
Refused 8
Blank 9 (M)

Health Conditions V2402 S9Q9A_2: EVER DIAGNOSED - MANIC-DEPRESSION, BIPOLAR DISORDER, OR MANIA
Location: 5392-5392 (width: 1; decimal: 0)
Interval: discrete
Question S9Q9A_2: Have you ever been told by a mental health professional, such as a psychiatrist or psychologist, that you had - Manic-depression, bipolar disorder, or mania
Question:
Value Label
1 Yes
2 No
7 Don't know
8 Refused
9 (M) Blank

Health Conditions	V2404	S9Q9A_4: EVER DIAGNOSED - POST-TRAUMATIC STRESS DISORDER<br>Location: 5394-5394 (width: 1; decimal: 0)<br>Interval: discrete<br>Question: S9Q9A_4: Have you ever been told by a mental health professional, such as a psychiatrist or psychologist, that you had - Post-traumatic stress disorder<br>Label Value<br>Yes 1<br>No 2<br>Don't know 7<br>Refused 8<br>Blank 9 (M)
Health Conditions	V2405	S9Q9A_5: EVER DIAGNOSED - ANOTHER ANXIETY DISORDER, SUCH AS A PANIC DISORDER<br>Location: 5395-5395 (width: 1; decimal: 0)<br>Interval: discrete<br>Question S9Q9A_5: Have you ever been told by a mental health professional, such as a psychiatrist or psychologist, that you had - Another anxiety disorder, such as a panic disorder<br>Question:<br>Label Value<br>Yes 1<br>No 2<br>Don't know 7<br>Refused 8<br>Blank 9 (M)
Health Conditions	V2403	S9Q9A_3: EVER DIAGNOSED - SCHIZOPHRENIA OR ANOTHER PSYCHOTIC DISORDER<br>Location: 5393-5393 (width: 1; decimal: 0)<br>Interval: discrete<br>Question S9Q9A_3: Have you ever been told by a mental health professional, such as a psychiatrist or psychologist, that you had - Schizophrenia or another psychotic disorder<br>Question:<br>Value Label<br>1 Yes<br>2 No<br>7 Don't know<br>8 Refused<br>9 (M) Blank
Health Conditions	V2406	S9Q9A_6: EVER DIAGNOSED - A PERSONALITY DISORDER<br>Location: 5396-5396 (width: 1; decimal: 0)<br>Interval: discrete<br>Question S9Q9A_6: Have you ever been told by a mental health professional, such as a psychiatrist or psychologist, that you had - A personality disorder (such as an antisocial or borderline personality disorder)<br>Value Label<br>1 Yes<br>2 No<br>7 Don't know<br>8 Refused<br>9 (M) Blank
Final Weight	V2622	RPWTSTM0: FINAL WEIGHT (STATE MALE INMATES ONLY)<br>5843-5851 (width: 9; decimal: 4) Location:<br>discrete Interval:<br>RPWTSTM0: Final Weight (State male inmates only) Question:<br>Label Value<br>Blank 9999.9999<br>(M)
Replicate Weights	V2623 - V2831	RPWTSTM1: REPLICATE WEIGHT #1 (STATE MALE INMATES ONLY)<br>discrete Interval:<br>RPWTSTM1: Replicate Weight #1 - 209 (State male inmates only)

Source: See reference 12
